# The role of the miR1976/CD105/integrin αvβ6 axis in vaginitis induced by *Escherichia coli* infection in mice

**DOI:** 10.1038/s41598-019-50902-w

**Published:** 2019-10-08

**Authors:** Lisha Jiang, Lingling Zhang, Can Rui, Xia Liu, Zhiyuan Mao, Lina Yan, Ting Luan, Xinyan Wang, Ying Wu, Ping Li, Xin Zeng

**Affiliations:** 1 Women’s Hospital of Nanjing Medical University, Nanjing Maternity and Child Health Care Hospital, Nanjing, 210004 China; 2 Department of Obstetrics and Gynecology, The Second Hospital of Anhui Medical University, Anhui, 230601 China; 3 Department of Obstetrics and Gynecology, Jiangsu Taizhou People’s Hospital, Taizhou, 225300 China; 4 Department of Anatomy, Histology, and Embryology, Nanjing Medical University, Nanjing, 210004 China

**Keywords:** Infection, Bacterial infection

## Abstract

Vaginitis is very common among women, especially women of childbearing age, and is associated with significantly increased risk of preterm birth and pelvic inflammatory diseases. An imbalance in the vaginal flora, the primary cause of vaginitis, promotes the initiation and progression of vaginal infections. However, the responsible mechanisms are still poorly understood. Using a murine vaginitis model of *Escherichia coli* infection, we demonstrated that decreased expression of microRNA1976 and increased expression of CD105 and integrin αvβ6 were closely associated with the progression of vaginal infection. Importantly, we demonstrated for the first time that the microRNA1976/CD105/integrin αvβ6 axis regulates *E. coli*-mediated vaginal infection in mice, as evidenced by the finding that *E. coli*-induced vaginal infection was reversed by microRNA1976 overexpression and exacerbated by CD105 overexpression. The regulation of CD105 and integrin αvβ6 by microRNA1976 was further confirmed in a murine model of vaginitis with adenoviral vector treatment. Taken together, our data suggested that microRNA1976 negatively regulates *E. coli*-induced vaginal infection in mice at least in part by suppressing CD105 and integrin αvβ6 expression. These findings may provide new insight into the mechanisms of *E. coli*-induced vaginitis, identify a novel diagnostic biomarker and a potential therapeutic target for flora imbalance-associated vaginitis.

## Introduction

A healthy vaginal microbiota is considered important for maintaining vaginal mucosal homeostasis and vaginal health and for preventing infections^1^. A bacterial imbalance refers to a change in the quantity and quality various bacteria in the normal bacterial flora in a certain part of the body due to influences of the host and external environment. Destruction of or imbalance in the vaginal microbiota might result in numerous urogenital diseases, including aerobic vaginitis and bacterial vaginosis^1^. Vaginitis, or vaginal infection, is prevalent in women and a common cause for which women seek medical care. Lactobacillus is the main bacteria of the normal vaginal flora. Bacterial imbalance leads to the reproduction of opportunistic pathogens, including bacteria, fungi and protozoa, which can cause vaginal diseases. Vaginitis is associated with a significantly increased risk of preterm birth, stillbirth and pelvic inflammatory diseases^2,3^ and has been reported to possibly contribute to uterine infection, cervical dysplasia, increased risk of postdelivery infections, and the acquisition and transmission of human immunodeficiency virus (HIV) and herpes simplex virus 2^2,4–6^. Although vaginitis, including aerobic vaginitis and bacterial vaginitis, has been studied for more than 15 years^7^, its underlying mechanisms are still poorly understood.

The maintenance of mucosal homeostasis, including that of the vaginal, oral and intestinal mucosae, involves various regulatory networks to thwart threats such as microbes and bacteria^8^. Recently, accumulating evidence has demonstrated that microRNAs (miRNAs) are pivotal in controlling these regulatory networks, helping to maintain mucosal barrier integrity and counterbalancing infections and inflammatory responses^9–11^. Numerous studies have shown that miRNAs play a critical role in regulating mucosal infection and mucosal inflammation in some mucosal diseases, such as those in the oral, intestinal, oesophageal and nasal mucosae^12–17^. However, the role of miRNAs in vaginal infections induced by an imbalance in the flora and the maintenance of mucosal homeostasis remains unclear. Therefore, our current study was designed to explore the role of miRNAs in vaginitis caused by *Escherichia coli* (*E. coli*) infection.

MiR1976 (MIMAT0009451), 20 nucleotides in length, is located at chromosome 1p36.11^18^. Publications have shown that miR1976 can function as a tumour suppressor and serve as a prognostic indicator for non-small cell lung cancer^18,19^. MiR1976 was suggested to be a competent predictor of the overall survival of patients with uterine corpus endometrial carcinoma, and has also been reported to be linked to certain bovine infectious diseases^20,21^. However, the effect of miR1976 on inflammation regulation and vaginal infection remains undetermined. Our previous study found that the expression of miR-1976 was significantly lower in mucosal tissues of women with vaginitis than in those of healthy women. Therefore, the present study aimed to investigate the functional roles of miR1976 in vaginitis caused by *E. coli* infection and to further explore the underlying mechanisms.

Bioinformatics analysis showed that the transforming growth factor receptor CD105 (endoglin) is a target gene of miR1976. CD105, a member of the transforming growth factor β (TGF-β) receptor family, is closely related to vaginal infection^22^. The TGF-β pathway is involved in regulating the signalling network of infections and inflammatory responses, including in vaginal candidiasis, chronic colitis and spinal neuroinflammation^23–25^. Several miRNAs, such as miR-499, miR-148b, miR-24 and miR-122, can bind with some elements of the TGF-β signalling pathway, including TGF-β receptor 1, Smad 2 and Smad 4^26–28^. Moreover, evidence has shown that CD105 plays an important role in angiogenesis, vascular disease, inflammation, infection, etc.^29–31^. For example, Unger *et al*.^32^ found that CD105 was associated with adverse pregnancy outcomes caused by malaria infection. Xiaobo *et al*.^33^ revealed the importance of miR-149-5p in the pathogenesis of preeclampsia by regulating CD105. In addition, some miRNAs, such as miR-1287 and miR-342-5p, have been reported to regulate CD105 and play an important role in angiogenesis, cell proliferation and osteogenic potential^34,35^. However, the function of CD105 in vaginal infections caused by *E. coli* remains unclear.

Colonization of mucosal surfaces is a critical initial step in most mucosal bacterial infections. Integrins are reported to promote effective mucosal colonization and represent potential targets for the prevention or treatment of bacterial infections^22^. By interacting with integrins, bacteria or viruses promote the adhesion of host cells to the basement membrane, which in turn causes or exacerbates mucosal infections^36,37^. One mechanism for protecting mucosal membranes from bacterial infection is the rapid renewal and exfoliation of mucosal epithelial cells. Kim *et al*.^37^ found that bacterial colonization and adhesion were inhibited when integrin-linked kinase was defective. Another study found that the ability of *group B Streptococcus* to invade epithelial cells is related to integrin αv^38^. Vaginal infection promoted the CD105 expression and integrin activity, while transduction with wild-type CD105 increased integrin β1 activity in human vaginal epithelial cells^22^. Rossi *et al*.^39^ found that the interaction between CD105 and integrin α5β1 plays a regulatory role in inflammation. Here, we aimed to verify whether miR1976, a potential treatment target for vaginitis, plays an important role in *E. coli*-induced vaginitis by regulating the CD105/integrin axis.

The objective was to investigate the role of the miR1976/CD105/integrin αvβ6 axis in vaginal infection caused by *E. coli*. We found that the expression of miR1976 was obviously decreased in vaginal tissues infected with *E. coli*. In contrast, *E. coli* infection significantly increased the levels of CD105 and integrin αvβ6. Adenoviral overexpression experiments indicated that overexpression of miR1976 inhibits the expression of CD105 and integrin αvβ6 and reverses *E. coli*-induced vaginal infection in mice. Collectively, these results suggest that miR1976 negatively regulates *E. coli*-induced vaginal infection in mice at least in part by inhibiting CD105 and integrin αvβ6. These findings provide new insight into the pathogenesis of vaginitis caused by *E. coli* infection and may open new avenues for the treatment and prevention of vaginitis.

## Results

### Vaginal infusion of *E. coli* induced vaginal infection in mice

**Figure 1:**
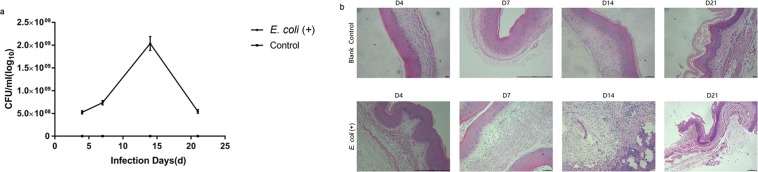
Vaginal infusion of *E. coli* induced vaginal infection in mice. (**a**) The bacterial burden in vaginal lavage fluids was assessed longitudinally on days 4, 7, 14, and 21; (**b**) Histopathology in reproductive tract tissues from mice after *E. coli* infection.

To establish the murine model of *E. coli* vaginal infection, the vaginas of female NIH mice were infected with 1 × 10^10^ CFUs of *E. coli* for 4, 7, 14 and 21 days. Neither the behaviour of infected mice nor the amount or volume of faeces and urine was changed after induction of vaginitis. Although the body weight did not differ between the control group and the experimental group, mice in the experimental group had relatively sparse, scruffy and dull coats (Supplementary Fig. S1). However, characteristics of human vaginitis, such as vaginal redness and swelling, were found in *E. coli*-infected mice (Supplementary Fig. S1b). Analysis of the direct vaginal lavage fluid obtained from the mice via Gram staining of colonies revealed Gram-negative cocci (*E. coli*) (Supplementary Fig. S2). At 4, 7, and 14 days after *E. coli* infection, the total number of vaginal microbes in the vaginal washes gradually increased and decreased by 21 days (Fig. 1a). In addition, histological analysis showed that *E. coli* infection caused epithelial damage, increased stratification of the epithelium, abundant sloughing of the epithelial mucosa, and an increased number of pus cells and parabasal epitheliocytes, especially 14 days after infection (Fig. 1b). Moreover, we observed neutrophils in the lamina propria and submucosa 14 days after *E. coli* infection. These results demonstrated that vaginal infusion of *E. coli* induces vaginal infection in mice. The collective symptoms of vaginitis were most obvious 14 days after *E. coli* infection. Therefore, based on the above results, we established the observation time point in subsequent experiments as 14 days after *E. coli* infection.

### *E. coli* infection regulated the expression of miR1976, CD105 and integrin αvβ6 in mouse vaginal tissues

**Figure 2:**
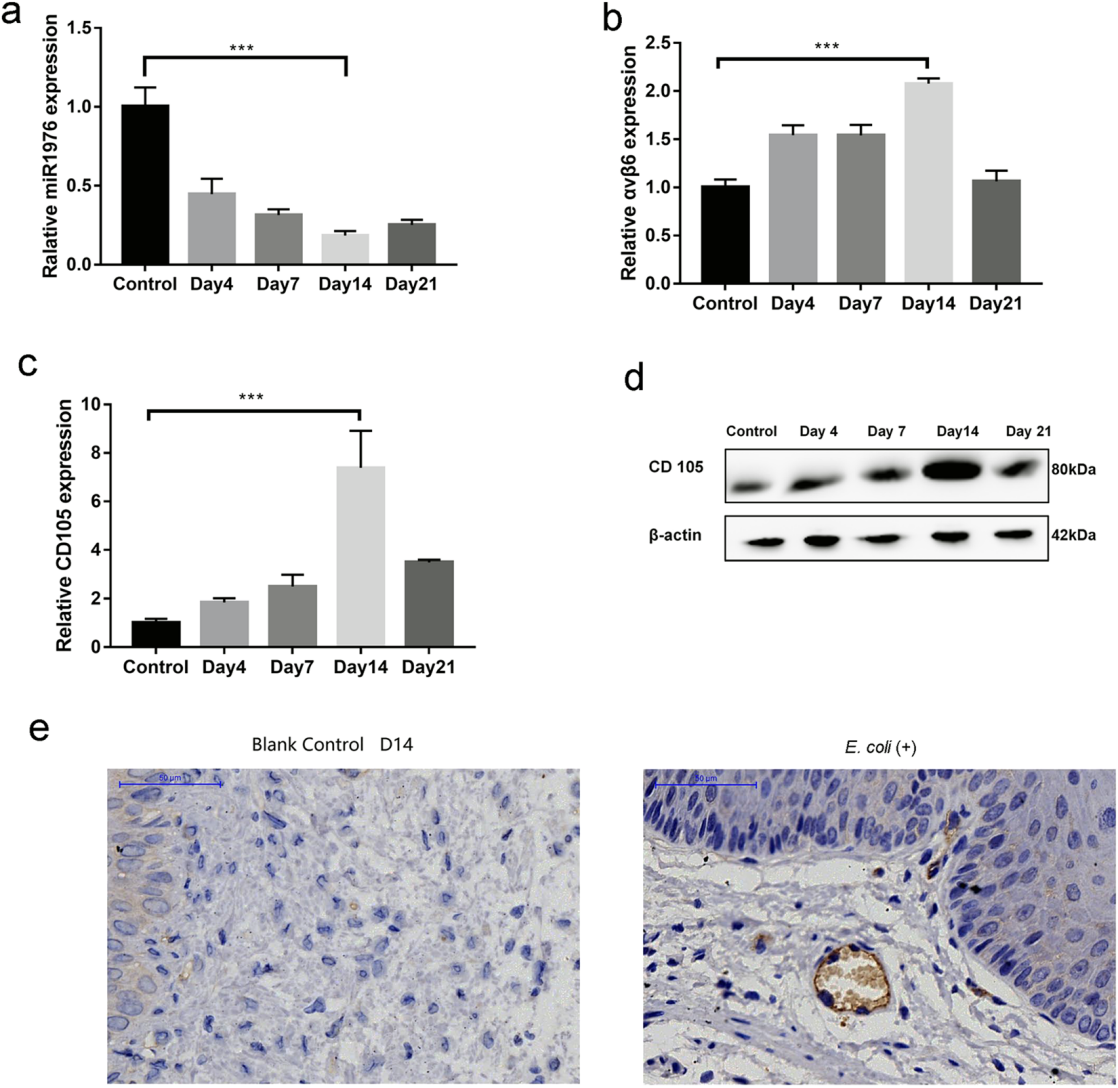
*E. coli* infection induced abnormal expression of miR1976, CD105 and integrin β6. (**a**) *E. coli* infection reduced the expression level of miR1976; (**b**) *E. coli* infection elevated the mRNA level of integrin αvβ6 in the vaginal tissue of mice; (**c**) CD105 protein expression was increased in the vaginal tissue of mice after *E. coli* infection, as shown by Western blot analysis; (**d**) The mRNA level of CD105 in the vaginal tissue of mice was increased after *E. coli* infection; (**e**) *E. coli* infection increased CD105 protein expression, as shown by immunofluorescence staining. *P < 0.05, **P < 0.01, compared with the control group.

In the present study, we investigated whether *E. coli* infection induced abnormal expression of miR1976 in mouse vaginal tissues. *E. coli* infection downregulated the expression level of miR1976 in mouse vaginal tissues (Fig. 2a). The transforming growth factor receptor CD105 is closely related to vaginal infections. Vaginal infection is reported to promote CD105 expression and integrin activity. To determine whether *E. coli*-induced vaginal infection in mice is associated with CD105 and integrin αvβ6 activation, the expression levels of CD105 and integrin αvβ6 were measured. Under normal conditions, the expression level of αvβ6 is very low, but it increases rapidly upon activation, as observed under wounding or inflammatory conditions^40^. *E. coli* infection elevated the mRNA levels of CD105 and integrin αvβ6 (Fig. 2b,d). Moreover, Western blot analyses and immunohistochemical staining revealed similar changes in the protein level of CD105 (Fig. 2c,e).

### Overexpression of miR1976 reversed *E. coli*-induced vaginal infection in mice via the CD105/integrin αvβ6 axis

**Figure 3:**
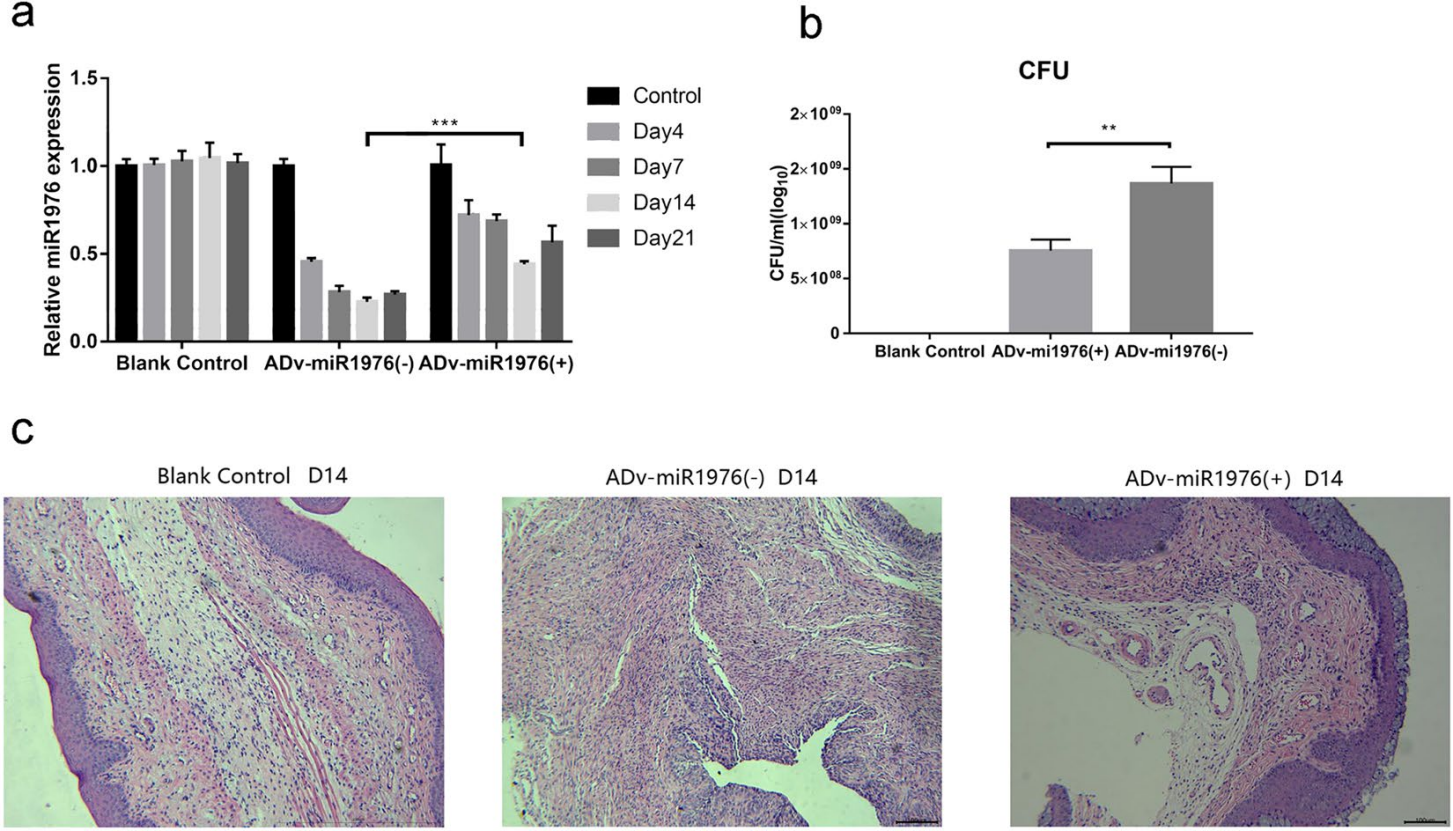
MiR-1976 overexpression reversed *E. coli*-induced vaginal infection in mice. (**a**) Administration of the miR-1976 overexpression vector restored the miR1976 expression suppressed by *E. coli* infection in the vaginal tissues of mice, as measured by qRT-PCR analysis; (**b**) The total number of vaginal microbes was significantly reduced after administration of the miR1976 adenoviral vector. (**c**) Histological analysis showed that abnormal pathological changes caused by *E. coli* can be reversed by miR1976 overexpression. *P < 0.05, **P < 0.01, compared with the control group; **P < 0.01, compared with the *E. coli* infection group.

As the above results revealed that *E. coli*-induced vaginal infection in mice was associated with miR1976 suppression, we further determined the role of miR1976 in *E. coli*-induced vaginal infection in mice. Adenoviral vectors overexpressing miR1976 were delivered into mice by multiple injections into the vaginal muscle layer, and the mice were infected with *E. coli*. Fluorescence imaging of green fluorescent protein (GFP) showed the successful transfection of mouse vaginal tissues with the miR1976 adenoviral vector (Supplementary Fig. S3). In addition, miR1976 expression, which was decreased in mouse vaginal tissues by *E. coli* infection, was restored by the delivery of the miR1976 adenoviral vector. The level of miR1976 in the *E. coli* + ADV-miR1976 (+) group was 191% that in the *E. coli* + ADV-miR1976 (−) group on day 14 (Fig. 3a). After administration of the miR1976 adenoviral vector, vaginal redness was decreased (Supplementary Fig. S4), and the total number of vaginal microbes in the vaginal washes was significantly reduced (Fig. 3b). In addition, H&E staining showed that overexpression of miR1976 attenuated vaginal infection caused by *E. coli* infection in mice (Fig. 3c). These results indicated that miR1976 may play an important role in *E. coli*-induced vaginal infection in mice.

**Figure 4:**
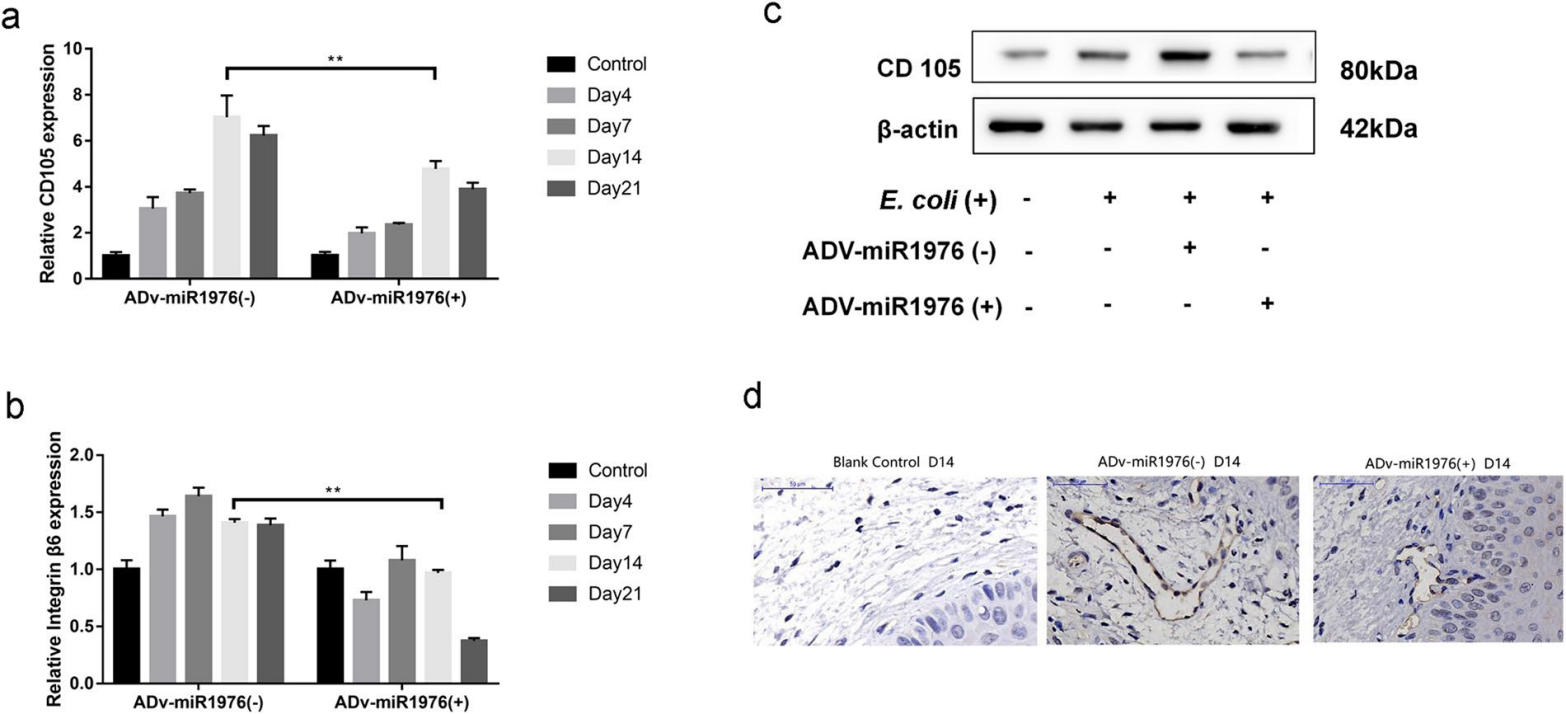
MiR1976 overexpression attenuated the alterations in CD105 and integrin αvβ6 expression. (**a**) Transfection of the miR1976 overexpression vector ameliorated the *E. coli* infection-induced increase in the mRNA expression level of CD105; (**b**) MiR1976 overexpression attenuated the *E. coli* infection-induced increase in the mRNA level of integrin αvβ6; (**c**) MiR1976 overexpression ameliorated the *E. coli*-induced alteration in CD105 expression. (**d**) MiR1976 overexpression attenuated the *E. coli* infection-induced increase in CD105 protein expression, as shown by immunohistochemical analysis. *P < 0.05, **P < 0.01, compared with the control group; * *P* < 0.05, **P < 0.01, compared with the *E. coli* infection group.

Our bioinformatics analysis and previous work identified CD105 as the target of miR1976. Consistent with the previous studies, ectopic expression of miR1976 decreased the *E. coli*-induced increases in the mRNA levels of CD105 and integrin αvβ6 (Fig. 4a,b). Western blot analysis showed that miR1976 overexpression ameliorated the *E. coli*-induced alteration in CD105 protein expression (Fig. 4c). Moreover, immunohistochemical staining showed that miR1976 overexpression decreased CD105 expression in mouse vaginas (Fig. 4d). These results suggested that miR1976 may play a negative regulatory role in *E. coli*-induced vaginal infection by affecting the expression of CD105 and integrin αvβ6.

### The CD105/integrin αvβ6 axis plays a key role in *E. coli*-induced vaginal infection

**Figure 5:**
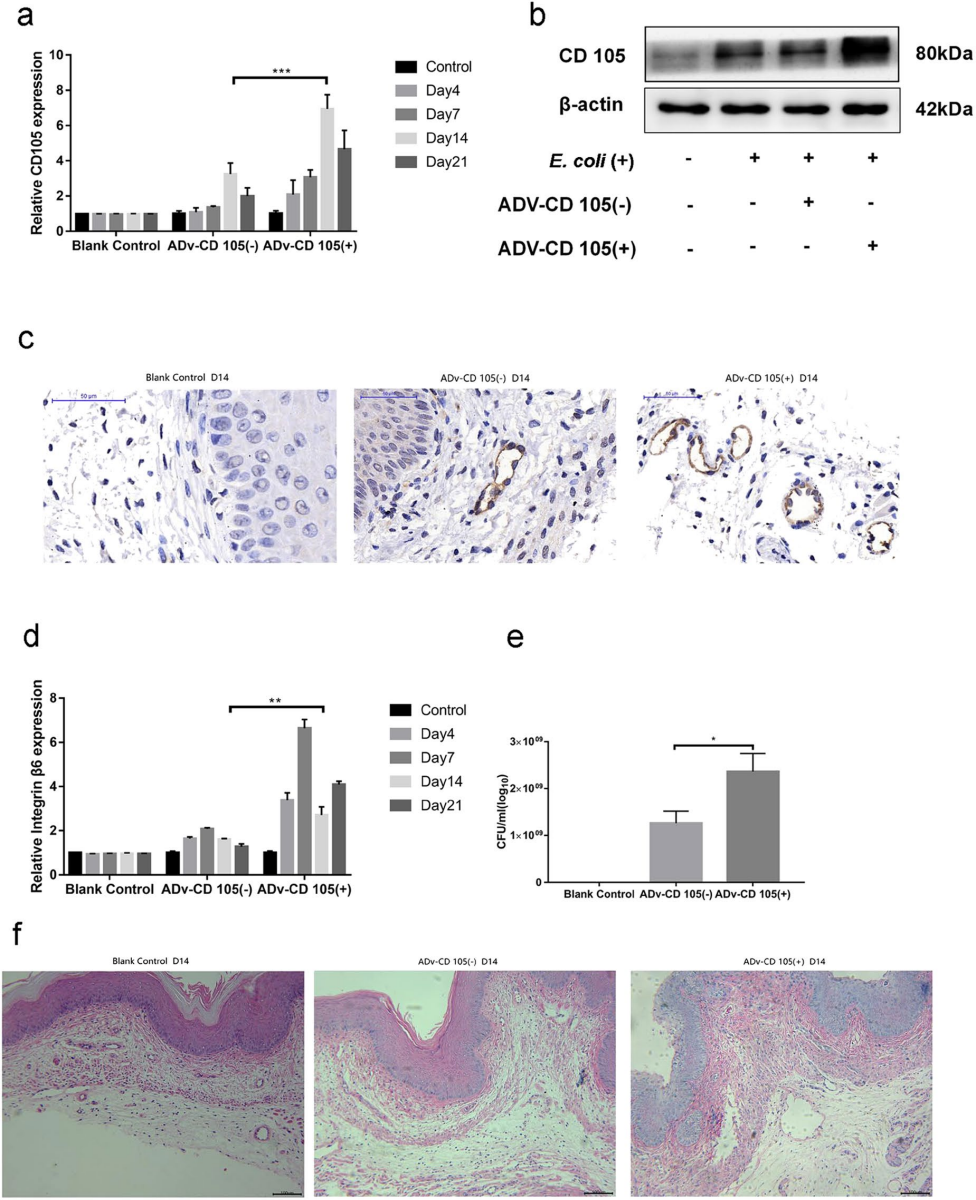
Overexpression of CD105 exacerbated vaginal infection in mice. (**a**) Administration of the CD105 overexpression adenoviral vector increased the mRNA expression level of CD105 in the vaginal tissue of mice; (**b**) CD105 overexpression increased the protein expression of CD105; (**c**) The change in CD105 protein expression after administration of the CD105 overexpression adenoviral vector was shown by immunohistochemical staining; (**d**) Administration of the CD105 adenoviral vector enhanced the mRNA level of integrin αvβ6; (**e**) The total number of vaginal microbes was also increased; (**f**) Haematoxylin-eosin staining showed that overexpression of CD105 exacerbated vaginal infection in mice. *P < 0.05, **P < 0.01, compared with the control group; *P < 0.05, **P < 0.01, compared with the *E. coli* infection group.

To further determine whether miR1976 directly regulates vaginal infection via CD105, mice received an adenoviral vector overexpressing CD105 by injections into the vaginal muscle layer and were simultaneously infected with *E. coli*. GFP fluorescence imaging indicated the successful transfection of mouse vaginal tissues with the CD105 adenoviral vector (Supplementary Fig. S5). As expected, administration of the CD105 adenoviral vector significantly elevated both the mRNA and protein (Fig. 5a,b) expression levels of CD105 in mouse vaginal tissues. In addition, immunohistochemical staining showed that CD105 overexpression increased CD105 expression in mouse vaginas (Fig. 5c). Moreover, administration of the CD105 adenoviral vector enhanced the mRNA expression of integrin αvβ6 in mouse vaginal tissues, as determined by qRT-PCR analyses (Fig. 5d). Overexpression of CD105 exacerbated vaginal infection in mice, as indicated by the noticeably increased oedema in mouse vaginas (Supplementary Fig. S6) and the significantly increased number of vaginal microbes (Fig. 5e). Furthermore, H&E staining showed that overexpression of CD105 exacerbated *E. coli*-induced vaginal infection in mice (Fig. 5f). The level of miR1976 in the *E. coli* + CD105 (−) group was 272% that in the *E. coli* + CD105 (+) group on day 14 (Supplementary Fig. S7).

**Figure 6:**
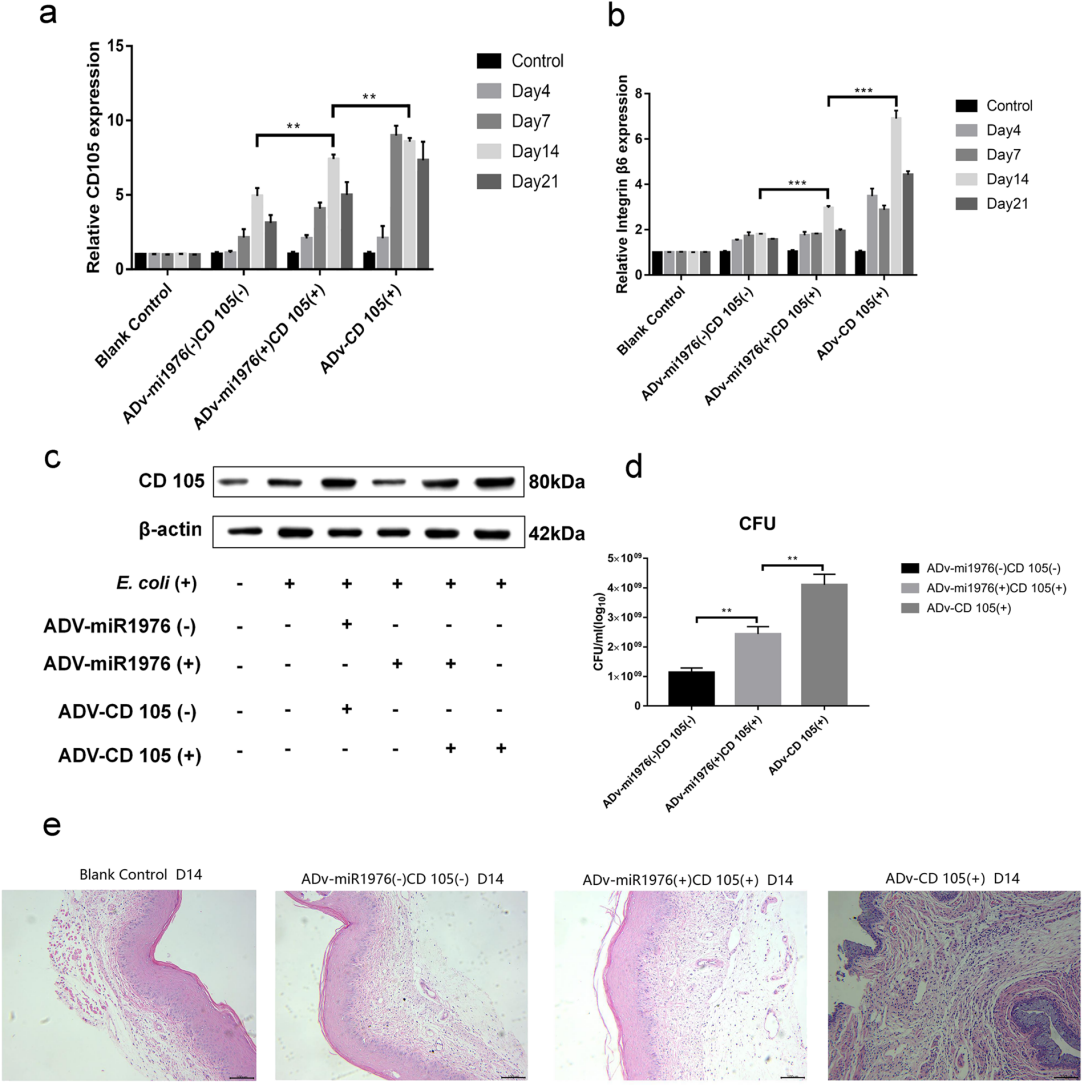
Simultaneous overexpression of miR1976 and CD105 ameliorated the deterioration of vaginal infection induced by CD105 overexpression. (**a**) Effects of miR1976 and CD105 overexpression on CD105 mRNA expression; (**b**) The change in the integrin αvβ6 mRNA level in the vaginal tissue of mice; (**c**) The change in CD105 protein expression after administration of the miR1976 and CD105 overexpression adenoviral vectors; (**d**) The number of vaginal microbes in the CD105 and miR1976 combination group was significantly lower than that in the CD105 adenoviral vector group; (**e**) Histological analysis indicated the alleviation of vaginal infection in the CD105 and miR1976 combination group compared with that in the CD105 adenoviral vector group.

To further verify the role of the CD105/integrin αvβ6 axis in *E. coli*-induced vaginal infection in mice, mice were simultaneously administered adenoviral vectors overexpressing miR1976 and CD105 and infected with *E. coli*. The expression levels of CD105 and αvβ6 in mouse vaginal tissues were measured (Fig. 6a–c). The level of αvβ6 in the *E. coli* + ADV-miR1976 (+) and CD105 (+) group was 160% that in the *E. coli* + ADV-miR1976 (−) and CD105 (−) group on day 14(Fig. 6b).The number of vaginal microbes in the CD105 and miR1976 combination group was significantly reduced compared with that in CD105 virus group (Fig. 6d). The H&E staining results showed greater alleviation of *E. coli*-induced vaginal infection in the CD105 and miR1976 combination group than in the CD105 adenoviral vector group (Fig. 6e).

Collectively, the above results suggested that the inhibitory effect of miR1976 on *E. coli*-induced vaginal infection may arise from direct regulation of the CD105 and integrin αvβ6 signalling pathways. Our study thus demonstrated that the miR1976/CD105/integrin αvβ6 axis plays an important role in *E. coli*-induced vaginal infection.

## Discussion

In this study, we reported decreased expression of miR1976 in vaginal tissues of mice infected with *E. coli*. MiR1976 downregulation was closely associated with flora imbalance-induced vaginal infection. We demonstrated that miR1976 overexpression reversed aspects of *E. coli*-induced vaginal infection, including vaginal redness, the number of vaginal microbes and pathological progression. MiR1976 exerted its anti-infective effect in vaginal infections in mice by inhibiting the expression of its target gene CD105 and its downstream integrin αvβ6. The identification of the miR1976/CD105/integrin αvβ6 signalling axis thus adds new evidence for the important roles of miRNAs in vaginal infection and provides new targets for the diagnosis, prognosis and therapy of vaginal infections.

The microbiome, an intricate ecosystem containing different microbial communities, varies substantially among tissues (e.g. vaginal, urethral, oral, nasal, gastrointestinal) and organs^41,42^. In recent years, our knowledge of the vaginal microbiome has improved considerably^41,43^. Lactic acid bacteria are the main bacteria in healthy vaginal flora, and under normal conditions, a balance is maintained among various bacteria. Any imbalance in the bacterial flora may result in vaginal infections, such as bacterial vaginitis, aerobic vaginitis, atrophic vaginitis, Candida vaginitis and Trichomonas vaginitis^41,44^. *E. coli* causes urinary tract infections and adverse pregnancy outcomes and is a commensal organism in the human gut^45^. Reports indicate that 24–31% of pregnant women and 9–28% of nonpregnant women are colonized by *E. coli*^46^. *E. coli* is the most common gram-negative pathogen, with an infection rate of 23% among symptomatic women with aerobic vaginitis^7^. The clinical risk factors for and underlying mechanisms of *E. coli* infection in the female vaginal tract are incompletely understood. Thus, this study explored the mechanism of *E. coli* in vaginal infection. We established the mouse model of vaginal infection described in a previous study^47^. Consistent with previous reports, the present study showed that exposure to *E. coli* induced vaginal infection in mice, as evidenced by apparent vaginal changes, including vaginal redness and swelling, abnormal secretion, and an increased number of vaginal microbes. Additional evidence was provided by the abnormal alterations seen in histopathological analysis, including an increase in the stratification of the epithelium, abundant sloughing of the epithelial mucosa, and the increased number of pus cells and parabasal epitheliocytes. These symptoms of vaginal infection were confirmed by a professional clinical pathologist. Taken together, our data revealed that vaginal infusion of *E. coli* induces vaginal infection in mice.

Studies have found that vaginal infections contribute to the progression of cervical dysplasia and increased risk of preterm birth, postpartum infections, pelvic inflammatory diseases, and viral infections^2,3,5,6^. Although vaginal infections have been studied for many years, the underlying mechanisms remain poorly understood. However, miRNAs are reported to play an important role in maintaining mucosal barrier integrity and counterbalancing mucosal infection and the inflammatory response^9–11^. Studies have shown that miRNAs are closely related to vaginal diseases and abnormal vaginal secretion^48–50^. In the present study, we found the first evidence that *E. coli*-induced vaginal infection is associated with suppression of miR1976 *in vivo*. We further showed that overexpression of miR1976 reversed *E. coli*-induced vaginal infection in mice, as evidenced by apparent changes in vaginal symptoms, including redness and oedema, abnormal secretion, and an increased number of vaginal microbes, as well as abnormal alterations seen in histopathological analysis. Few studies have addressed miR1976 and have generally focused on its use as a prognostic indicator for tumours or as a predictor for the overall survival of cancer patients^18,21^. Research on the role of miRNAs in vaginitis or vaginal infections is rare. Indeed, the results of this study provide the first indication that miR1976 negatively regulates *E. coli*-induced vaginal infection *in vivo*. These data may provide a scientific basis for the involvement of miRNAs in regulating vaginal infection and offer a new approach for the treatment of vaginal infections.

Evidence has suggested that miRNAs exert their biological functions by regulating the expression of target genes^51–53^. The TGF-β signalling pathway plays a critical role in the signalling network of infections and inflammatory responses. Several miRNAs that target elements of the TGF-β signalling pathway have been identified^26,27^. The transforming growth factor receptor CD105 is a member of the TGF-β receptor family and is closely related to angiogenesis, vascular disease, inflammation, and infections^22,29–31^. Moreover, evidence indicates that miRNAs such as miR-1287, miR-149-5p, and miR-342-5p play important roles in angiogenesis, preeclampsia, cell proliferation and osteogenic potential by regulating CD105^34,35^. However, the function of CD105 in *E. coli*-induced vaginal infections remains unclear. We identified CD105 as the target of miR1976 using bioinformatics analysis and a dual luciferase reporter assay (unpublished data). In this study, we found that *E. coli*-induced vaginal infection was associated with CD105 activation in a mouse model. Moreover, we showed that overexpression of miR1976 resulted in decreased expression of CD105 in the mouse model of vaginal infection. To further determine the regulatory role of the miR1976/CD105 axis in *E. coli*-induced vaginal infection in mice, mice were inoculated with an adenoviral vector overexpressing CD105 and infected with *E. coli*. CD105 overexpression exacerbated vaginal infection in mice, as indicated by the noticeably increased vaginal redness in the mice and the significantly increased number of vaginal microbes. In addition, H&E staining showed that overexpression of CD105 exacerbated vaginal infection in mice. Together, these findings suggest that the targeting of CD105 by miR1976 plays an important role in vaginal infection in mice. The results of this study may provide new research ideas for *E. coli* infection in other tissues, such as those of the intestines and bladder.

Colonization of mucosal surfaces is an important initial step in most mucosal bacterial infections. Rapid renewal and exfoliation of mucosal epithelial cells is a mechanism for protecting mucous membranes from bacterial infections and eliminating microbial infections. Integrins are reported to promote effective mucosal colonization and vaginal infections and are potential targets for the prevention or treatment of bacterial infections^22,54,55^. CD105 influences the synthesis of several members of the integrin family and regulates integrin-mediated cell adhesion^30^. Rossi *et al*.^39^ found that the interaction between CD105 and integrin α5β1 plays a regulatory role in inflammation. Vaginal infection promotes CD105 expression and integrin activity, and upregulation of CD105 enhances integrin β1 activity in normal human vaginal epithelial cells^22^. Thus, CD105 and integrin may play an important role in vaginal infection. However, the role of CD105/integrin αvβ6 in vaginal infection has not been reported. Our results showed that *E. coli*-induced vaginal infection promotes CD105 expression and integrin αvβ6 activity. For example, overexpression of miR1976 decreased the expression of integrin αvβ6 in a mouse model of vaginal infection. Furthermore, overexpression of CD105 enhanced the mRNA level of integrin αvβ6 in mouse vaginal tissues. In addition, our results suggested that simultaneous overexpression of miR1976 and CD105 reduced the deterioration of vaginal infection induced by CD105 overexpression. These results indicated that the miR1976/CD105/integrin αvβ6 axis plays an important role in *E. coli*-induced vaginal infection.

In summary, for the first time, the present study provides important evidence that miR1976 negatively regulates *E. coli*-related vaginal infection in mice in the *in vivo* setting. Importantly, we demonstrated that the miR1976/CD105/integrin αvβ6 axis plays a critical role in *E. coli*-induced vaginal infection in mice and that this pathway may affect the colonization and adhesion of *E. coli*. These new findings indicate the important role of the miR1976/CD105/integrin αvβ6 axis in *E. coli*-related vaginal infection and may provide a novel biomarker for the diagnosis and prognosis of vaginitis and offer a potential target for vaginitis therapy.

## Materials and Methods

See the Supplemental Material for further details concerning methods.

### Mice and the murine model of vaginal infection

The animal study was approved by the ethics committee of Nanjing Medical University, Nanjing, China. Female NIH mice were purchased from the Animal Research Center of Nanjing Medical University. Mice were handled in accordance with the recommendations in the guidelines of the Animal Care and Welfare Committee of Nanjing Medical University.

The mouse model of vaginal infection has previously been described^47,56–58^. After vaginal infection, vaginal lavage fluid was collected by flushing the vaginas with phosphate-buffered saline (PBS) and collecting the fluid in sterile tubes. Colony-forming units (CFUs) in 100 μl of vaginal lavage fluid were counted after being cultured in culture dishes at 37 °C for 24 h.

### *In vivo* delivery of adenoviral vectors

In a separate set of animal studies, adenoviral vectors (ADVs) were delivered into mice by injection into the vaginal muscle layer according to the manufacturer’s protocol. Mice were randomly divided into groups (n = 12 per group): the control group, *E. coli* group, *E. coli* + ADV-miR1976 group and *E. coli* + ADV-miR1976 + ADV-CD105 group. Following completion of the experiment, mice were sacrificed, and their vaginal tissues were collected for analysis.

### Histological analysis

Vaginal tissue sections were stained with haematoxylin and eosin (H&E) for observation of pathological changes.

### Quantitative reverse transcription-polymerase chain reaction

The levels of CD105, integrin αv-β6 and β-actin mRNA in mouse vaginal tissues were determined by quantitative reverse transcription-polymerase chain reaction (qRT-PCR). The primers used were as follows: β-actin-orward, 5′-TCAAGATCATTGCTCCTCCTGAG-3′ and reverse, 5′-ACATCTGCTGGAAGGTGGACA-3′; CD105-forward, 5′-AGGTCTCCGAGGGCTGTGTA-3′ and reverse, 5′-GTCTCCGTGCCATTTTGCT-3′; integrin αv-β-forward, 5′-GGTGGAACTGGAAGTGTTAGGG-3′ and reverse, 5′-GGAGCATTTCTTTTGGTGTGG-3′; MiR1976-forward, 5′-GCGGCCCTCCTGCCCTCC-3′

and reverse, 5′-CAGCCACAAAAGAGCACAAT-3′.

### Western blot analysis

Proteins were extracted from mouse vaginal tissues. Western blot analyses were performed to determine protein expression levels.

### Immunohistochemical staining

Immunohistochemistry was performed to analyse the expression of CD105 in mouse vaginal tissues.

### Statistical analysis

Statistical analyses were performed with SPSS 16.0. All data are expressed as the means ± standard deviations. One-way ANOVA was used to compare significant differences among multiple groups. For comparisons between two groups, a *t* test was used. A value of *p* < 0.05 was considered significant.

## Supplementary information

Supplementary IInformation

Dataset

## Data Availability

All data generated or analysed during this study are included in this published article (and its Supplementary Information Files), are available from the corresponding author on reasonable request.

## References

[1] Kaambo E, Africa C, Chambuso R, Passmore JS (2018). Vaginal microbiomes associated with aerobic vaginitis and bacterial vaginosis. Front. Public Health.

[2] Mulu W, Yimer M, Zenebe Y, Abera B (2015). Common causes of vaginal infections and antibiotic susceptibility of aerobic bacterial isolates in women of reproductive age attending at Felegehiwot Referral Hospital, Ethiopia: a cross sectional study. BMC Womens Health.

[3] Kaambo E, Africa CWJ (2017). The threat of aerobic vaginitis to pregnancy and neonatal morbidity. Afr. J. Reprod. Health.

[4] Vornhagen J (2018). Group B streptococcus exploits vaginal epithelial exfoliation for ascending infection. J. Clin. Invest..

[5] Amatya R, Bhattarai S, Mandal PK, Tuladhar H, Karki BM (2013). Urinary tract infection in vaginitis: a condition often overlooked. Nepal Med. Coll. J..

[6] Deng, Z. L. *et al*. Metatranscriptome analysis of the vaginal microbiota reveals potential mechanisms for protection against metronidazole in bacterial vaginosis. *mSphere* **3**, 10.1128/mSphereDirect.00262-18 (2018). 10.1128/mSphereDirect.00262-18 PMC5990888 29875146

[7] Donders GGG, Bellen G, Grinceviciene S, Ruban K, Vieira-Baptista P (2017). Aerobic vaginitis: no longer a stranger. Res. Microbiol..

[8] Neudecker V, Yuan X, Bowser JL, Eltzschig HK (2017). MicroRNAs in mucosal inflammation. J. Mol. Med. (Berl.).

[9] Chassin C (2012). MicroRNA-146a-mediated downregulation of IRAK1 protects mouse and human small intestine against ischemia/reperfusion injury. EMBO Mol. Med..

[10] Dai X (2015). MicroRNA-193a-3p reduces intestinal inflammation in response to microbiota via down-regulation of colonic PepT1. J. Biol. Chem..

[11] Huang Z (2014). miR-141 regulates colonic leukocytic trafficking by targeting CXCL12beta during murine colitis and human Crohn’s disease. Gut.

[12] Arao TC, Guimaraes AL, de Paula AM, Gomes CC, Gomez RS (2012). Increased miRNA-146a and miRNA-155 expressions in oral lichen planus. Arch. Dermatol. Res..

[13] Otsuka-Tanaka Y (2013). Oral lining mucosa development depends on mesenchymal microRNAs. J. Dent. Res..

[14] Valmiki S, Ahuja V, Paul J (2017). MicroRNA exhibit altered expression in the inflamed colonic mucosa of ulcerative colitis patients. World J. Gastroenterol..

[15] Grassi A (2018). A coordinate deregulation of microRNAs expressed in mucosa adjacent to tumor predicts relapse after resection in localized colon cancer. Mol. Cancer.

[16] Fassan M (2017). Noncoding RNAs as drivers of the phenotypic plasticity of oesophageal mucosa. World J. Gastroenterol..

[17] Suojalehto H (2014). Altered microRNA expression of nasal mucosa in long-term asthma and allergic rhinitis. Int. Arch. Allergy Immunol..

[18] Chen G, Hu J, Huang Z, Yang L, Chen M (2016). MicroRNA-1976 functions as a tumor suppressor and serves as a prognostic indicator in non-small cell lung cancer by directly targeting PLCE1. Biochem. Biophys. Res. Commun..

[19] Yang C (2016). Integrative analysis of microRNA and mRNA expression profiles in non-small-cell lung cancer. Cancer Gene Ther..

[20] Gupta SK (2018). Detection of microRNA in cattle serum and their potential use to diagnose severity of Johne’s disease. J. Dairy Sci..

[21] Wang Y, Xu M, Yang Q (2019). A six-microRNA signature predicts survival of patients with uterine corpus endometrial carcinoma. Curr. Probl. Cancer.

[22] Muenzner P, Bachmann V, Zimmermann W, Hentschel J, Hauck CR (2010). Human-restricted bacterial pathogens block shedding of epithelial cells by stimulating integrin activation. Science.

[23] Jo H (2018). Regulatory dendritic cells induced by mesenchymal stem cells ameliorate dextran sodium sulfate-induced chronic colitis in mice. Gut Liver.

[24] Evangelista AF (2018). Bone marrow-derived mesenchymal stem/stromal cells reverse the sensorial diabetic neuropathy via modulation of spinal neuroinflammatory cascades. J. Neuroinflammation.

[25] Xie W (2018). Knockdown of MicroRNA-21 promotes neurological recovery after acute spinal cord injury. Neurochem. Res..

[26] Miscianinov V (2018). MicroRNA-148b targets the TGF-beta pathway to regulate angiogenesis and endothelial-to-mesenchymal transition during skin wound healing. Mol. Ther..

[27] Sun Y (2018). miR-24 and miR-122 negatively regulate the transforming growth factor-beta/smad signaling pathway in skeletal muscle fibrosis. Mol. Ther. Nucleic Acids.

[28] Wu J (2019). MiR-499 regulates myoblast proliferation and differentiation by targeting transforming growth factor beta receptor 1. J. Cell. Physiol..

[29] Ottaviano G (2015). Endoglin (CD105) expression in sinonasal polyposis. Eur. Arch. Otorhinolaryngol..

[30] Rossi E, Lopez-Novoa JM, Bernabeu C (2014). Endoglin involvement in integrin-mediated cell adhesion as a putative pathogenic mechanism in hereditary hemorrhagic telangiectasia type 1 (HHT1). Front. Genet..

[31] Koska, M. T. Hospital prenatal care programs are an ounce of prevention. *Hospitals* **64**, 50, 54, 57–59 (1990). 2307427

[32] Unger HW (2019). Sulphadoxine-pyrimethamine plus azithromycin may improve birth outcomes through impacts on inflammation and placental angiogenesis independent of malarial infection. Sci. Rep..

[33] Xiaobo Z, Qizhi H, Zhiping W, Tao D (2019). Down-regulated miR-149-5p contributes to preeclampsia via modulating endoglin expression. Pregnancy Hypertens.

[34] Ishiy FAA (2018). CD105 is regulated by hsa-miR-1287 and its expression is inversely correlated with osteopotential in SHED. Bone.

[35] Yan, X. C. *et al*. miR-342-5p is a notch downstream molecule and regulates multiple angiogenic pathways including notch, vascular endothelial growth factor and transforming growth factor beta signaling. *J. Am. Heart Assoc*. **5**; 10.1161/jaha.115.003042 (2016). 10.1161/JAHA.115.003042 PMC4802463 26857067

[36] Xicohtencatl-Cortes J (2007). Intestinal adherence associated with type IV pili of enterohemorrhagic *Escherichia coli* O157:H7. J. Clin. Invest..

[37] Kim M (2009). Bacteria hijack integrin-linked kinase to stabilize focal adhesions and block cell detachment. Nature.

[38] de Gaetano GV (2018). The *Streptococcus agalactiae* cell wall-anchored protein PbsP mediates adhesion to and invasion of epithelial cells by exploiting the host vitronectin/alphav integrin axis. Mol. Microbiol..

[39] Rossi E (2018). Human endoglin as a potential new partner involved in platelet-endothelium interactions. Cell. Mol. Life Sci..

[40] Chen X (2011). Intestinal epithelial cell-derived integrin alphabeta6 plays an important role in the induction of regulatory T cells and inhibits an antigen-specific Th2 response. J. Leukoc. Biol..

[41] Wang ZL (2016). Diagnosis and microecological characteristics of aerobic vaginitis in outpatients based on preformed enzymes. Taiwan J. Obstet. Gynecol..

[42] Ding T, Schloss PD (2014). Dynamics and associations of microbial community types across the human body. Nature.

[43] Tempera G, Furneri PM (2010). Management of aerobic vaginitis. Gynecol. Obstet. Invest..

[44] Sun X, Qiu H, Jin Y (2017). Highly efficient treatment of aerobic vaginitis with simple acidic buffered gels: the importance of pH and buffers on the microenvironment of vaginas. Int. J. Pharm..

[45] Cools P (2017). The role of *Escherichia coli* in reproductive health: state of the art. Res. Microbiol..

[46] Kim YA, Lee K, Chung JE (2018). Risk factors and molecular features of sequence type (ST) 131 extended-spectrum-beta-lactamase-producing *Escherichia coli* in community-onset female genital tract infections. BMC Infect. Dis..

[47] Pascual L, Ruiz F, Giordano W, Barberis IL (2010). Vaginal colonization and activity of the probiotic bacterium *Lactobacillus fermentum* L23 in a murine model of vaginal tract infection. J. Med. Microbiol..

[48] Silva SS, Lopes C, Teixeira AL, de Sousa MJC, Medeiros R (2015). Forensic miRNA: potential biomarker for body fluids?. Forensic Sci. Int. Genet..

[49] Liu X (2014). Differential expression of microRNAs in periurethral vaginal wall tissues of postmenopausal women with and without stress urinary incontinence. Menopause.

[50] Sirker M, Fimmers R, Schneider PM, Gomes I (2017). Evaluating the forensic application of 19 target microRNAs as biomarkers in body fluid and tissue identification. Forensic Sci. Int. Genet..

[51] Li X (2018). Downregulation of miR-218-5p promotes invasion of oral squamous cell carcinoma cells via activation of CD44-ROCK signaling. Biomed. Pharmacother..

[52] Hong W, Zhang P, Wang X, Tu J, Wei W (2018). The effects of MicroRNAs on key signalling pathways and epigenetic modification in fibroblast-like synoviocytes of rheumatoid arthritis. Mediators Inflamm..

[53] Gomes A, da Silva IV, Rodrigues CMP, Castro RE, Soveral G (2018). The emerging role of microRNAs in aquaporin regulation. Front. Chem..

[54] Harrell CW, Dey J, Shamsi SA, Foley JP, Warner IM (1998). Enhanced separation of antidepressant drugs using a polymerized nonionic surfactant as a transient capillary coating. Electrophoresis.

[55] Goode D (2014). HSV-2-driven increase in the expression of alpha4beta7 correlates with increased susceptibility to vaginal SHIV(SF162P3) infection. PLoS Pathog..

[56] Mosci P (2013). Mouse strain-dependent differences in estrogen sensitivity during vaginal candidiasis. Mycopathologia.

[57] Pietrella D (2011). Beneficial effect of *Mentha suaveolens* essential oil in the treatment of vaginal candidiasis assessed by real-time monitoring of infection. BMC Complement. Altern. Med..

[58] Luna-Tapia A (2015). ERG2 and ERG24 are required for normal vacuolar physiology as well as *Candida albicans* pathogenicity in a murine model of disseminated but not vaginal candidiasis. Eukaryot. Cell.

