# Supplementary material for: The role of the miR1976/CD105/integrin αvβ6 axis in vaginitis induced by Escherichia coli infection in mice
Source: Sci Rep. 2019 Oct 8;9:14456. doi: 10.1038/s41598-019-50902-w (PMC6783613; doi:10.1038/s41598-019-50902-w)
Supplement: Supplementary file 1 — Supplementary IInformation [file 41598_2019_50902_MOESM1_ESM.docx]

**The role of the miR1976/CD105/integrin αvβ6 axis in vaginitis induced by *Escherichia coli* infection in mice**

Lisha Jiang^1,2,#^, Lingling Zhang^1,#^, Can Rui^1^, Xia Liu^3^, Zhiyuan Mao^4^, Lina Yan^1^, Ting Luan^1^, Xinyan Wang^1^, Ying Wu^1^, Ping Li^1,*^, Xin Zeng^1,*^

**Supplementary Fig. S1.** Overview of mice after infection. (**a**) Changes in the body weights of mice after *E. coli* infection; (**b**) Vulvas appeared red and swollen after *E. coli* infection.

**Supplementary Fig. S2.** *E. coli* was observed in the **vaginal** lavage fluid of infected mice using Gram staining.

**Supplementary Fig. S3.** GFP ﬂuorescence imaging of mouse vaginal tissues after treatment with the miR1976 adenoviral vector.

**Supplementary Fig. S4.** The miR-1976 overexpression vector attenuated the redness and swelling of the vaginal tissue.

**Supplementary Fig. S5.** GFP ﬂuorescence imaging of mouse vaginal tissues after treatment with the CD105 adenoviral vector.

**Supplementary Fig. S6.** The severity of vaginal redness and swelling was increased after treatment with the CD105 overexpression adenoviral vector.

**Supplementary Fig. S7.** The effect of CD105 overexpression on the expression level of miRNA1976.

**Supplementary Fig. S8.** Based on the editor's comments, we edited the figure and organized the original Western blotting strips. Since we cut the PVDF membrane according to the molecular weight of the target protein, the Western blotting strips are separately dispersed. We showed the original Western blotting strips as follows.

**Supplementary information**

**Materials and methods**

***Chemicals and reagents***

β-Oestradiol was purchased from Sigma-Aldrich (St. Louis, MO), and castor oil was obtained from Aladdin (Shanghai, China). *Escherichia coli* (*E. coli*)  strain CLT 5034 was provided by the Microbiological Laboratory of Nanjing Drum Tower Hospital and grown on Sabouraud dextrose agar at 37℃. For the preparation of *E. coli* suspensions, the strain was inoculated in Luria-Bertani (LB) medium at a ratio of 2:100,  shaken at 220 rpm and 37℃ for 12h and cryopreserved in 15% glycerinum at -80℃. Colonies were counted using a hemocytometer, and *E. coli* suspensions were adjusted to the desired concentration with LB medium.

Primary antibodies against CD105 and β-actin were obtained from Cell Signaling Technology (Beverly, MA). Bulge-loopTM miRNA qRT-PCR Primers for both miR1976 and U6 were designed by RiboBio (Guangzhou, China). The primers used for CD105, integrin β6 and β-actin were synthesized by Invitrogen (Carlsbad, CA). The sources of other materials are noted accordingly in the text.

***Mice and*** ***murine vaginal infection model***

Female NIH mice (6-8 weeks) were purchased from the Animal Research Center of Nanjing Medical University. Mice were housed in polypropylene cages, received a normal diet with access to water ad libitum, and were maintained on a 12 h fluorescent light/12 h darkness cycle at a room temperature of 22 ± 0.5℃. Mice were handled in accordance with the recommendations in the guidelines of the Animal Care and Welfare Committee of Nanjing Medical University. Our study protocol was approved by the Committee on the Ethics of Animal Experiments of Nanjing Medical University.

The mouse model of vaginal infection has previously been described^32-35^. Mice received 0.1 mg of β-oestradiol in 100 μl of castor oil three days before and on the day of infection. Mice in the control group were injected subcutaneously with 100 µl of castor oil. β-Oestradiol was injected once weekly until the end of the experiment. Mice were anaesthetized with isoflurane and inoculated vaginally with 1×10^10^ colony-forming units (CFUs) of *E. coli* in 20 μl of sterile PBS. After inoculation, mice were kept positioned with the vagina facing upwards for more than 5 min to reduce leakage. On the 4th day, 7th day, 14th day and 21st day after vaginal inoculation, after the vaginas were flushed with 100 μl of sterile phosphate-buffered saline (PBS) and washed 10 times, vaginal lavage fluid was collected in a 1.5 ml sterile Eppendorf tube. A series of 10-fold serial dilutions was made using sterile PBS and inoculated in LB solid agar culture dish. CFUs were counted after culture at 37°C for 24 h and are expressed as CFU/100 μl of vaginal lavage fluid.

**In vivo *delivery of adenoviral vectors***

In a separate set of animal studies, ADVs were delivered into mice by injection into the vaginal muscle layer. Briefly, each mouse was injected at three points in the vaginal myometrium (10 µl per injection point) with an adenoviral vector (2×10^10^ plaque-forming units (PFUs)). Mice were randomly divided into groups (n=12 per group): the control group; *E. coli* group, in which mice were infected with *E. coli*; *E. coli*+ADV-control group, in which mice were administered negative control adenoviral vector and infected with *E. coli*; *E. coli*+ADV-miR1976 group, in which mice were administered the adenoviral vector expressing miR1976 and were infected with *E. coli*; *E. coli*+ADV-CD105 group, in which mice were administered the adenoviral vector overexpressing CD105 and were infected with *E. coli*; and *E. coli*+ADV-miR1976+ADV-CD105 group, in which mice were administered adenoviral vectors overexpressing miR1976 and CD105 and were infected with *E. coli*. Following the completion of the experiment, mice were sacrificed, and their vaginal tissues were collected for analysis.

***Histological analysis***

Vaginal tissues were fixed with 10% formalin, dehydrated and embedded in paraffin, and thin sections (4 μm) were prepared. Subsequently, sections were deparaffinized with xylene and rehydrated in water through a graded series of alcohols. Vaginal tissue sections were stained with haematoxylin and eosin (H&E). Slides were observed using a Carl Zeiss research microscope (Dublin, CA) and confirmed by a clinical pathologist.

***Quantitative reverse transcription-polymerase chain reaction (qRT-PCR)***

Total RNA was isolated from vaginal tissues by Trizol reagent (Invitrogen, CA) according to the manufacturer’s instructions. RNA for miRNA analysis was isolated by the GeneJET RNA purification method (Thermo Fisher Scientific, MA). Two micrograms of total RNA was reverse transcribed to cDNA using Professional TRIO Reverse Transcriptase (BIOMETRA, Germany). RT-PCR was performed using Power SYBR Green Master Mix (TaKaRa, Japan) and a Quant Studio 6 Flex real-time PCR detection system (Thermo Fisher Scientific, MA). The primers used were as follows:

miR1976—forward, 5'-GCGGCCCTCCTGCCCTCC-3' and reverse, 5'-CAGCCACAAAAGAGCACAAT-3';

β-actin—forward, 5'-TCAAGATCATTGCTCCTCCTGAG-3' and reverse,

5'-ACATCTGCTGGAAGGTGGACA-3';

CD105—forward, 5'-AGGTCTCCGAGGGCTGTGTA-3' and reverse, 5'-GTCTCCGTGCCATTTTGCT-3';

and integrin αv-β6—forward, 5'-GGTGGAACTGGAAGTGTTAGGG-3' and reverse, 5'-GGAGCATTTCTTTTGGTGTGG-3'.

The mRNA expression levels for each gene were normalized to the corresponding level of U6 or β-actin. Fold changes in gene expression were calculated by the comparative threshold cycle (Ct) method using the formula 2^-ΔΔCt^.

***Western blot analysis***

Vaginal tissues were homogenized in lysis buffer and were then centrifuged at 4°C for 15-20 min. Protein concentrations were measured with a BCA Protein Assay (Beyotime Biotechnology, Shanghai, China). Equal amounts of protein (40 μg) were fractionated via SDS-PAGE and transferred to polyvinylidene fluoride (PVDF) membranes (Millipore, Billerica, MA). PVDF membranes were blocked with 5% non-fat milk and probed with anti-CD105 and anti-β-actin antibodies overnight at 4°C. After washing with Tris-buffered saline/Tween 20 three times, membranes were incubated with horseradish peroxidase-conjugated secondary antibodies at room temperature for 1 h. Target proteins were subsequently visualized using enhanced chemiluminescence.

***Immunohistochemical staining***

Immunohistochemistry was performed according to the reported method. Briefly, 4-μm serial paraffin-embedded vaginal tissue sections were de-waxed in xylene and rehydrated in an alcohol gradient. Endogenous peroxidase activity was quenched by exposure to 3% (v/v) hydrogen peroxide solution for 15 min at room temperature. Vaginal tissue sections were boiled in citrate buffer (pH 6.0, 10 mM) for antigen retrieval and incubated with 5% (w/v) bovine serum albumin (BSA) to block non-specific binding. Sections were incubated with the primary antibody at 4°C and subsequently incubated with biotinylated secondary antibody. Finally, vaginal tissue sections were visualized with 3,3'-diaminobenzidine and counterstained with haematoxylin.
