# Supplementary material for: The role of the miR1976/CD105/integrin αvβ6 axis in vaginitis induced by Escherichia coli infection in mice
Source: Sci Rep. 2019 Oct 8;9:14456. doi: 10.1038/s41598-019-50902-w (PMC6783613; doi:10.1038/s41598-019-50902-w)
Supplement: Supplementary file 2 — Dataset [file 41598_2019_50902_MOESM2_ESM.pdf]

The role of the miR1976/CD105/integrin αvβ6 axis in vaginitis induced by Escherichia coli infection in mice

Lisha Jiang[1,2,#], Lingling Zhang[1,#], Can Rui[1], Xia Liu[3], Zhiyuan Mao[4], Lina Yan[1], Ting Luan[1], Xinyan Wang[1], Ying Wu[1], Ping Li[1,*], Xin Zeng[1,*]

Figure S1

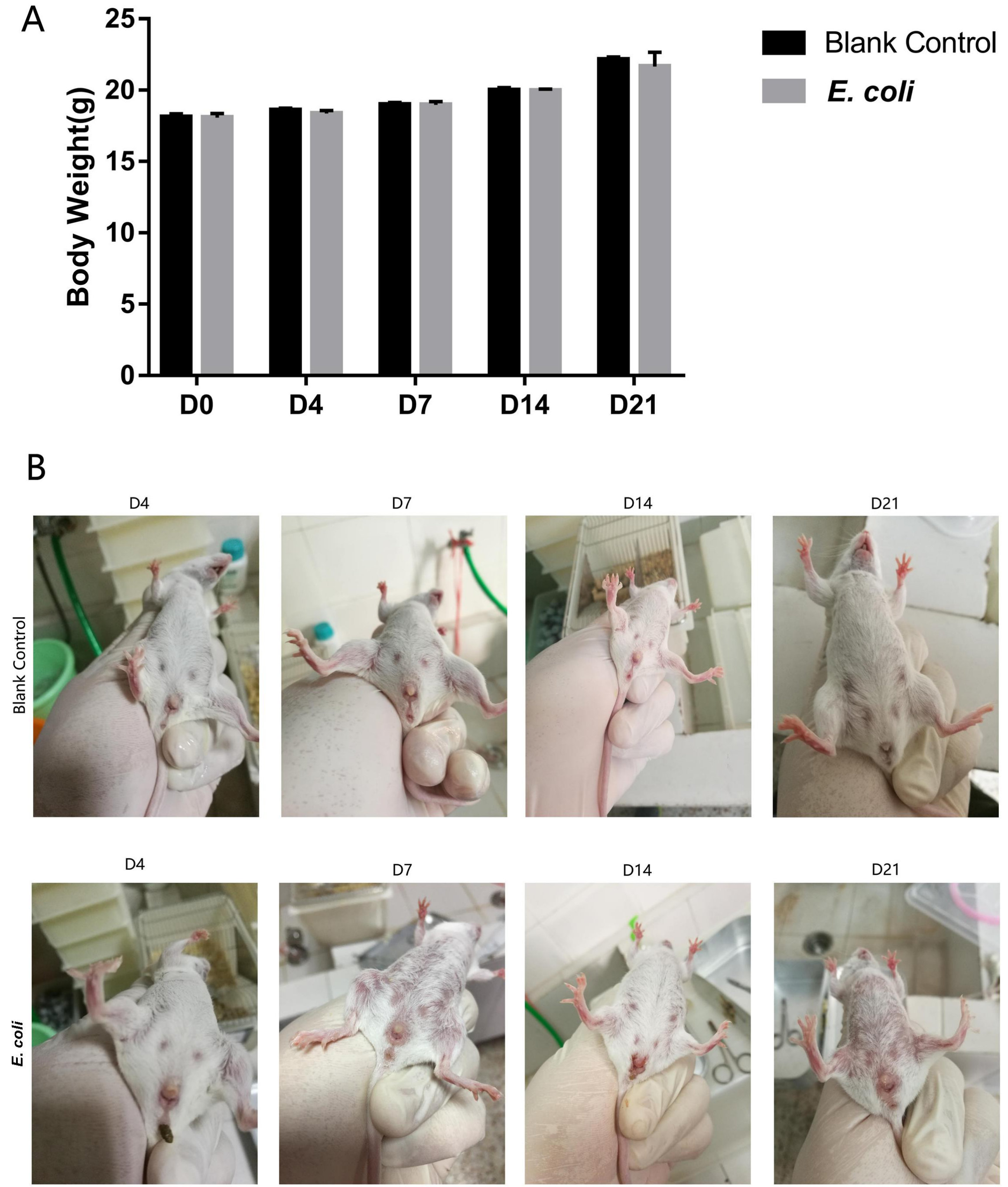
A
Body Weight(g)
0
5
10
15
20
25
D0
D4
D7
D14
D21
Blank Control
E. coli
B
D4
D7
D14
D21
Blank Control
E. coli

Figure S2

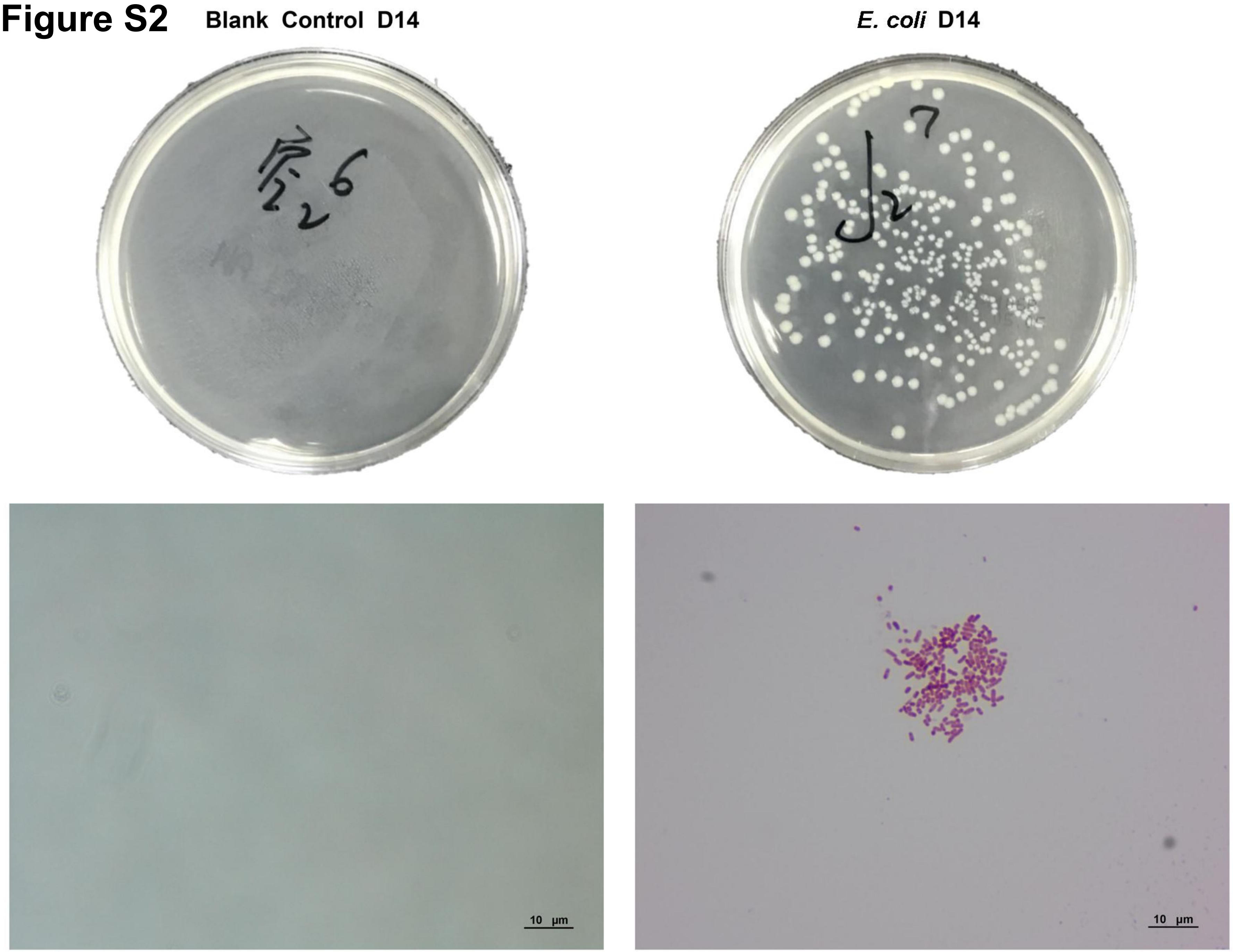

Figure S3

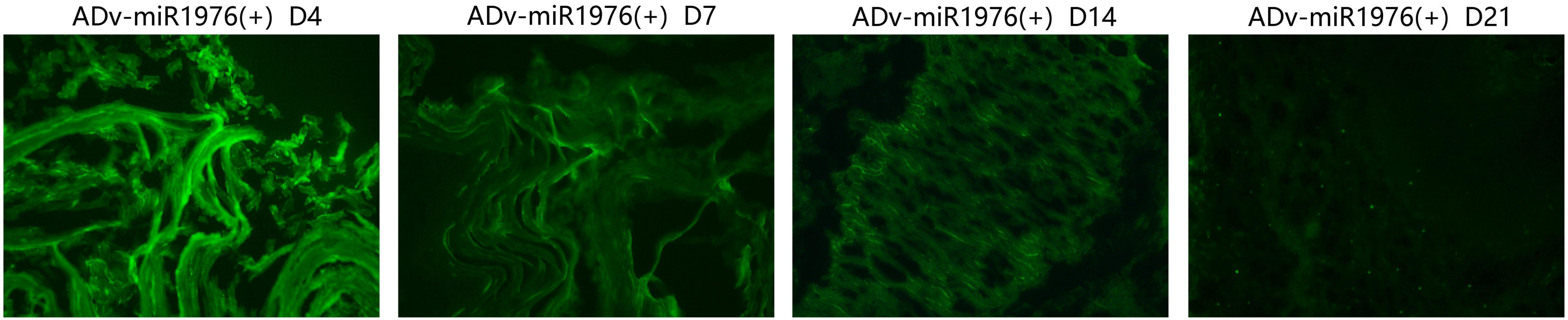
ADv-miR1976(+) D4
ADv-miR1976(+) D7
ADv-miR1976(+) D14
ADv-miR1976(+) D21

Figure S4

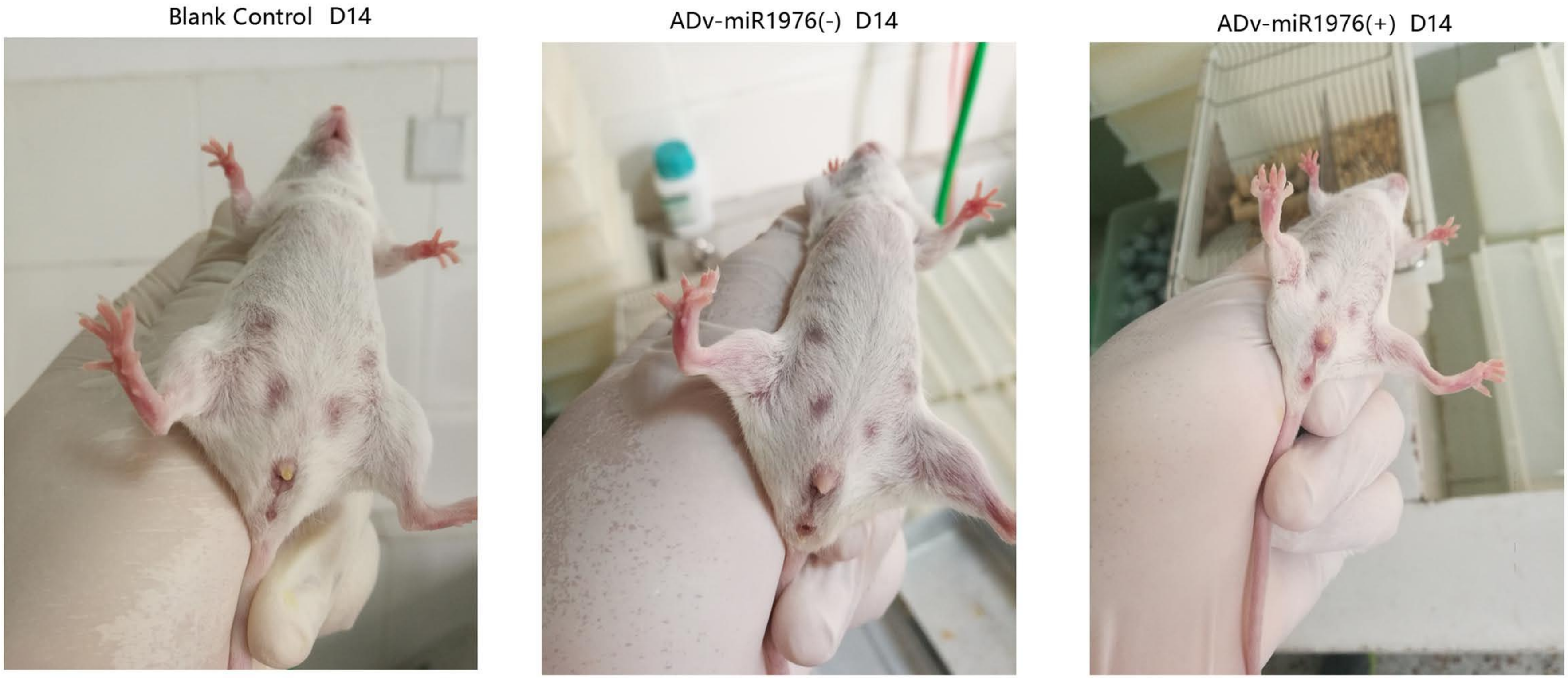

Figure S5

ADv-CD 105(+) D4 | ADv-CD 105(+) D7 | ADv-CD 105(+) D14 | ADv-CD 105(+) D21

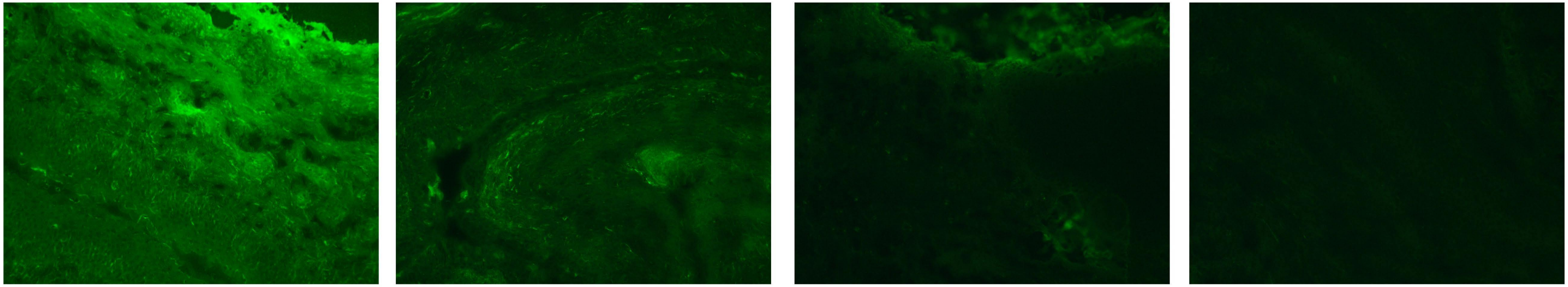

Figure S6

Blank Control D14

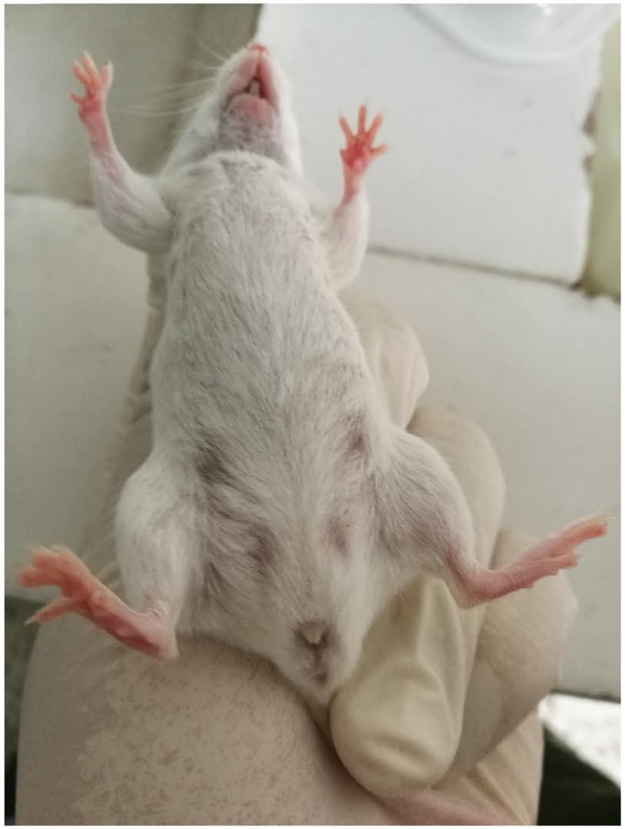

ADv-CD 105(-) D14

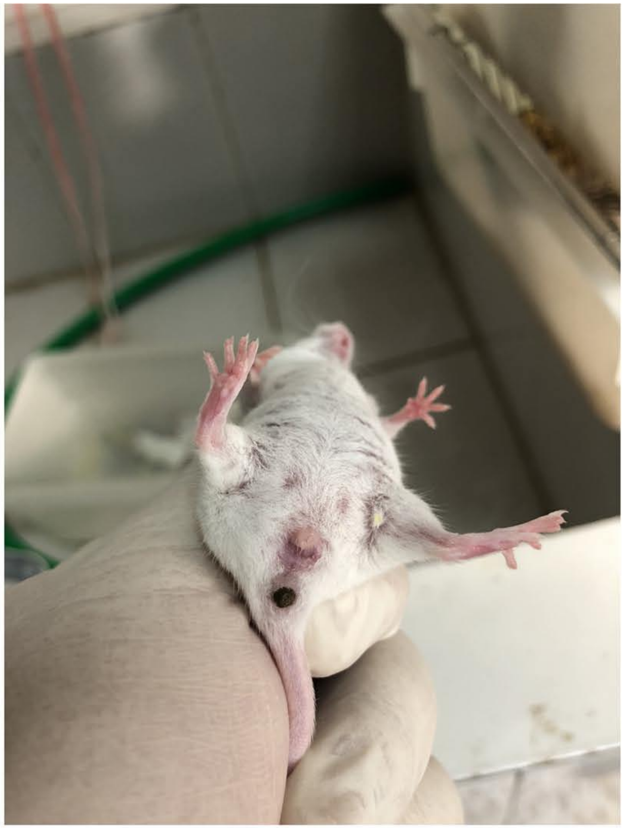

ADv-CD 105(+) D14

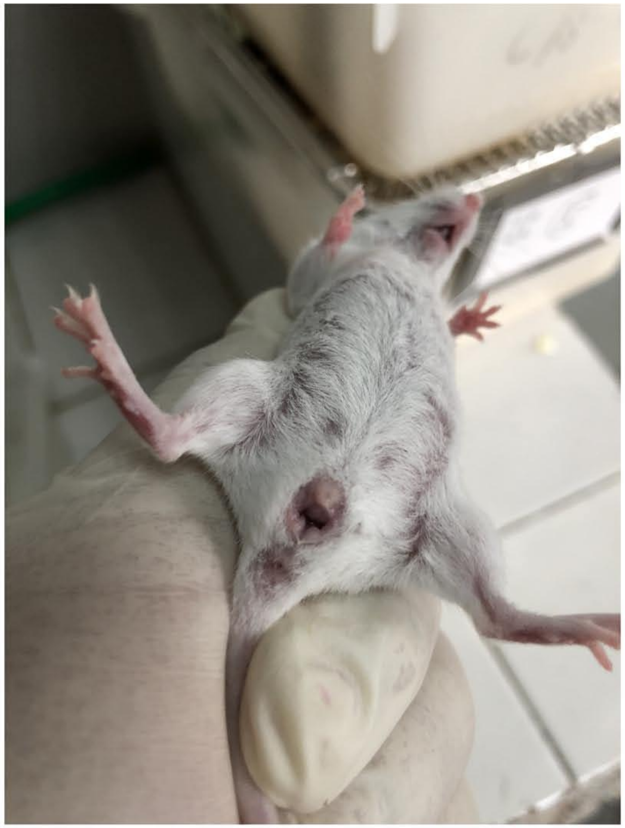

Figure S7

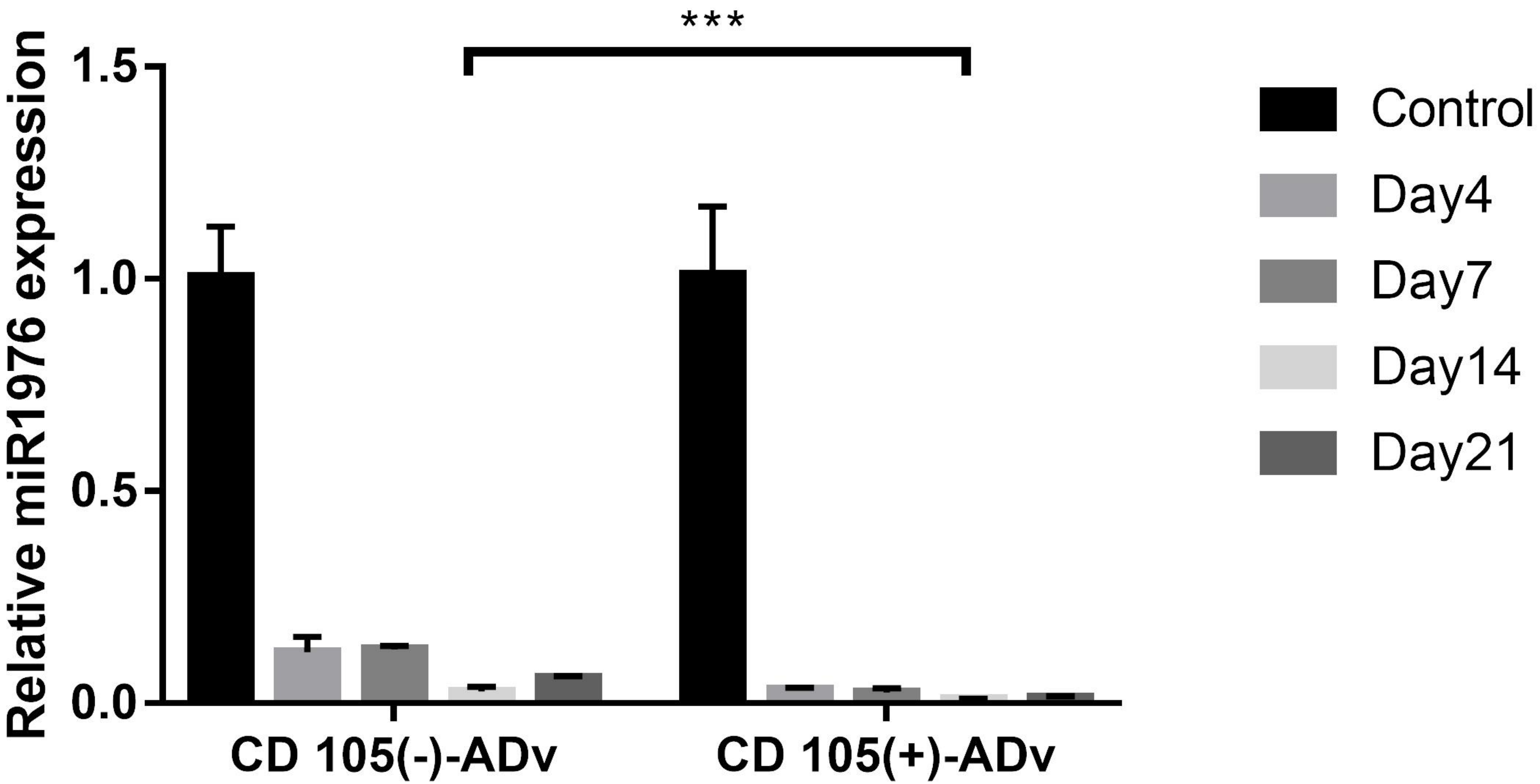
***
Relative miR1976 expression
1.5
1.0
0.5
0.0
CD 105(-)-ADv
CD 105(+)-ADv
Control
Day4
Day7
Day14
Day21

Figure S8

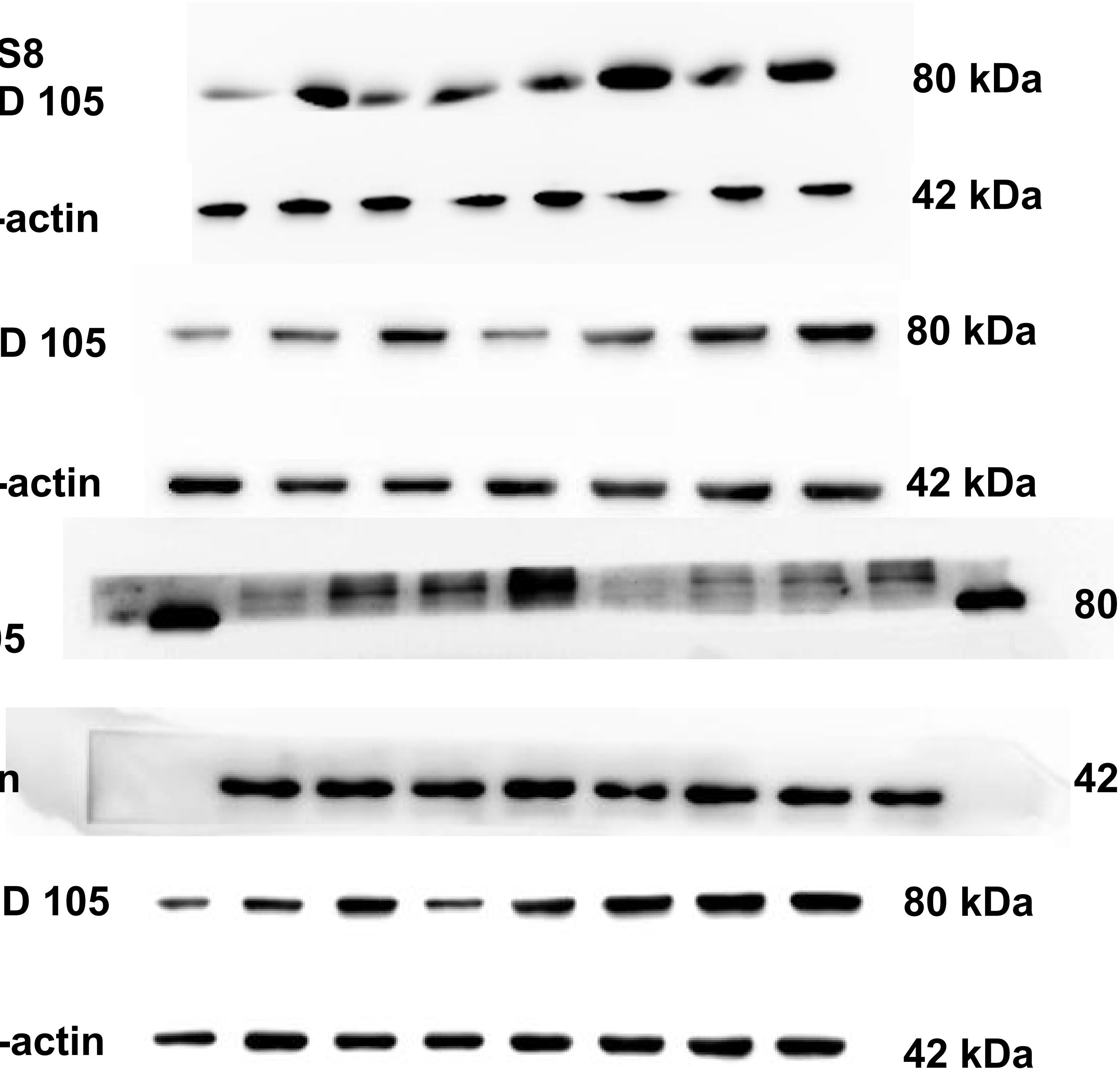
CD 105
80 kDa
β-actin
42 kDa
CD 105
80 kDa
β-actin
42 kDa
CD 105
80 kDa
β-actin
42 kDa
CD 105
80 kDa
β-actin
42 kDa
